# Relationship Between Oxidative Stress and Cardiovascular Risk in Adolescents in Montenegro

**DOI:** 10.3390/ijms26157650

**Published:** 2025-08-07

**Authors:** Aleksandra Klisic, Marija Bozovic, Barbara Ostanek, Janja Marc, Paschalis Karakasis, Filiz Mercantepe, Jelena Kotur-Stevuljevic

**Affiliations:** 1 Faculty of Medicine, University of Montenegro, 81000 Podgorica, Montenegro; 2 Center for Laboratory Diagnostics, Primary Health Care Center, 81000 Podgorica, Montenegro; 3 Institute of Public Health of Montenegro, 81000 Podgorica, Montenegro; 4 Department of Clinical Biochemistry, Faculty of Pharmacy, University of Ljubljana, 1000 Ljubljana, Sloveniajanja.marc@ffa.uni-lj.si (J.M.); 5 Second Department of Cardiology, Aristotle University of Thessaloniki, General Hospital “Hippokration”, 54642 Thessaloniki, Greece; pakar15@hotmail.com; 6 Department of Endocrinology and Metabolism, Faculty of Medicine, Recep Tayyip Erdogan University, Rize 53100, Turkey; filizmercantepe@hotmail.com; 7 Department for Medical Biochemistry, Faculty of Pharmacy, University of Belgrade, 11000 Belgrade, Serbia

**Keywords:** antioxidants, atherosclerosis, cardiovascular risk, inflammation, oxidative stress

## Abstract

The pathophysiological mechanism linking oxidative stress and cardiovascular disease (CVD) is not completely elucidated, especially in young individuals. This study aimed to examine redox status in an adolescent Montenegrin population in relation to cardiovascular risk score (CVRS). A cohort of 182 adolescents (76% girls) aged between 16 and 19 was examined. Total antioxidant status (TAS), superoxide dismutase (SOD), advanced oxidation protein products (AOPPs), malondialdehyde (MDA), and total oxidant status (TOS) were determined. Pro-oxy score, anti-oxy score, and oxy score were calculated as comprehensive parameters of overall redox homeostasis status. CVRS was calculated by summarizing several risk factors (i.e., sex, age, obesity, hypertension, dyslipidemia, impaired fasting glucose, and smoking). A significant positive correlation between CVRS and TOS (rho = 0.246, *p* = 0.001) and AOPP (rho = 0.231, *p* = 0.002) and MDA (rho = 0.339, *p* < 0.001), respectively, and a negative correlation with the TAS/TOS ratio (rho= −0.208, *p* = 0.005) was observed. An increase in pro-oxy scores as well as oxy scores with CVRS risk increase were observed. Anti-oxy scores did not differ between CVRS subgroups. There is a significant relationship between cardiovascular risk score and oxidative stress in the adolescent Montenegrin population. These findings support the possibility for improvement of age-specific CVD risk algorithms by adding redox homeostasis parameters in addition to conventional ones.

## 1. Introduction

Cardiovascular disease (CVD) is a multifactorial complex disease and a major culprit for disability and death worldwide [1]. Although CVD is rare in childhood, the manifestation of its risk factors often tracks from a young age, especially in children living with obesity [2,3]. Obesity is an independent risk factor for CVD [4]. According to global estimates, more than 150 million adolescents aged 10 to 19 (i.e., one in eight teenagers) will suffer from obesity by 2030 [5]. It is also concerning that the trend of CVD incidence is on the rise in teenagers between the ages of 15 and 19, particularly among girls [2]. Considering the rapid increase in the prevalence of obesity in the adolescent population, which is caused by a sedentary lifestyle and unhealthy diet, it is necessary to pay special attention to the screening and stratification of cardiovascular risk in youngsters [6]. Young persons with prominent CVD risk factors, such as obesity and its related comorbidities, such as hypertension, dyslipidemia, insulin resistance, and liver steatosis, due to the unfavorable cumulative effect of these risk factors over time [2,3], are of particular interest.

A large number of CVD risk prediction algorithms for the quantification of individual risk have been proposed and validated [7,8,9,10]. Since each algorithm is based on a particular population, its validity is constrained by sample size variability. Also, most of these tools are designed for older people [7,8,9,10]. However, the younger population, especially adolescents whose CVD incidence trend is on the rise, is neglected regarding quantification and stratification of CVD risk [2,11].

Oxidative stress and inflammation are regarded as accelerators of endothelial dysfunction, causing increased endothelial permeability, decreased nitric oxide (NO) bioavailability, and compromised vasodilation that lead to a pathological cascade of subclinical atherosclerosis [1]. Lipid peroxidation is regarded as the earliest step in this process. Low-density lipoprotein (LDL) particles, especially small, dense LDL, are highly susceptible to reactive oxygen species (ROS)-induced modification [12]. During this process, both its lipid and protein content become oxidatively modified, enabling the accumulation of cholesterol in subendothelial macrophages and enhancing the consequent proinflammatory immune response, which is the hallmark of subclinical atherosclerosis [12,13]. Studies show that early signs of inflammation and oxidative stress, which underlie endothelial dysfunction and atherosclerosis, are present at a younger age, even though manifest CVD is not yet present [14,15].

Secondary products of lipid and protein oxidation have been examined in recent years to assess their utility in cardiometabolic disorders [15,16]. Indeed, children with obesity had higher levels of oxidized LDL prior to the increase in carotid intima–media thickness (cIMT), an indicator of subclinical atherosclerosis [17]. Hence, it is of great importance to identify young people with a high CVD risk and treat them in a timely manner. In this regard, diagnostic biomarkers that could increase the predictive and discriminatory ability of cardiovascular risk scores (CVRS) would be beneficial. We hypothesized that oxidative stress biomarkers may have the potential to improve CVRS stratification. In line with this, the aim of the present study is to evaluate CVRS in an adolescent Montenegrin cohort and to examine its association with redox status parameters. If this relationship is confirmed, the CVD risk algorithm may be upgraded by incorporating novel atherosclerosis-related parameters in addition to traditional ones.

## 2. Results

Table 1 showed basic parameters in subgroups given by CVRS risk: low, moderate, and high. The adolescents with the highest CVRS were preferentially male subjects and smokers, they had higher BMI, WC, hsCRP, blood pressure, lower HDL-c and higher TG/HDL-c ratio. Other measured parameters were not significantly different between CVRS subgroups.

Redox status parameters across CVRS risk subgroups were presented in Table 2.

A significantly higher frequency of high-risk subjects was found among males compared to females. Oxidative stress was higher in a high-risk group, i.e., TOS, AOPP, and MDA were significantly higher in this group compared to the low-risk group. Although neither antioxidant enzyme SOD nor TAS was different in CVRS risk subgroups, the TAS/TOS ratio representing antioxidant blood potency was significantly lower in both moderate- and high-risk groups compared to the low-risk group (Table 2).

**Figure 1:**
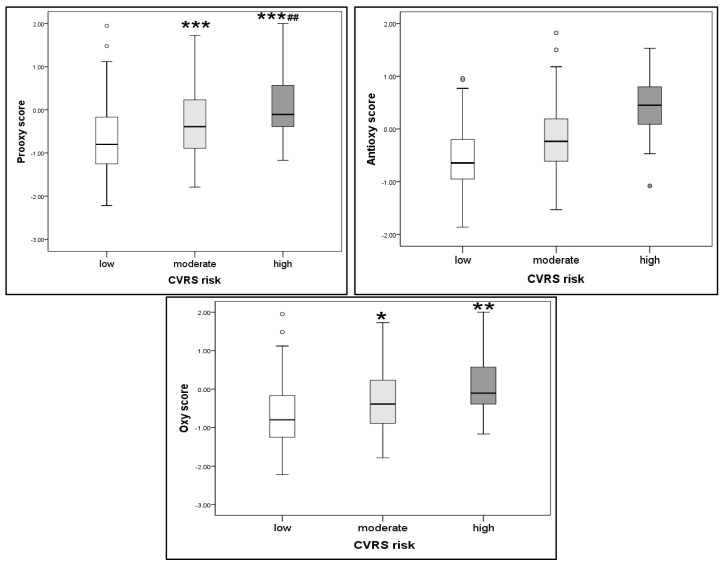
Redox scores according to CVRS risk score. *, **, *** *p* < 0.05, 0.01, 0.001; ## *p* < 0.01.

In order to obtain deeper insight into the relationship between oxidative stress and CVRS, we have additionally calculated redox scores from originally measured redox scores’ parameters: pro-oxy, anti-oxy, and oxy scores. The results are presented in Figure 1. While anti-oxy scores did not differ between CVRS subgroups, we noticed an increase in pro-oxy scores as oxy scores with CVRS risk increased and additionally confirmed the relationship between CVRS and redox status parameters.

Spearman’s correlation analysis also confirmed the relationship between CVRS and redox status elements. This analysis revealed a significant positive correlation between CVRS and TOS and AOPP and MDA, respectively, while there was a negative correlation with the TAS/TOS ratio (Table 3).

### Factorial (Principal Component Analysis)

**Figure 2:**
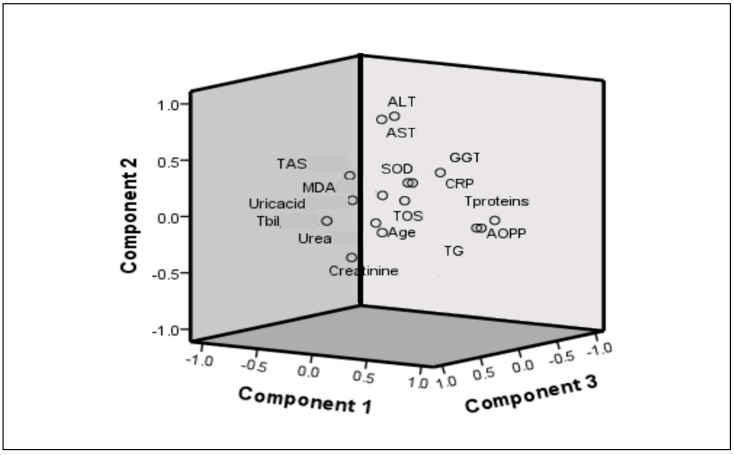
Component plot in rotated space from Principal Component Analysis (PCA).

Analysis adequacy is confirmed by the Kaiser–Meyer–Olkin (KMO) index of 0.563 (must be greater than 0.500) and Bartlett’s sphericity index (*p* < 0.001; the condition is *p* < 0.05). Initial analysis included urea, creatinine, uric acid, total proteins, triglycerides, AST, ALT, CRP, TAS, TOS, SOD, AOPP, and MDA. The detailed results of the factor analysis with varimax rotation are presented in Table 4 and in Figure 2. The total percentage of variability in this analysis is 41%, with Factor 1 comprising 16%, Factor 2: 13%, and Factor 3: 12%. Factor 1 included AOPP, TG, and TOS; Factor 2 consisted of ALT and AST; and Factor 3 included uric acid, creatinine, and TAS.

**Figure 3:**
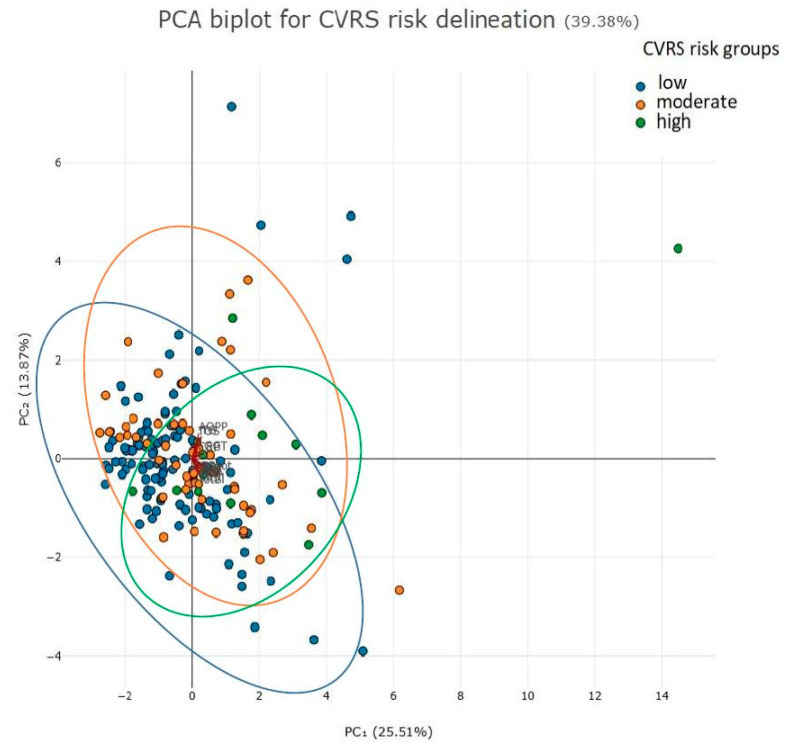
Principal Component Analysis (PCA) biplot of patients subgroups according to CVRS risk (low, moderate, high).

Figure 3 illustrates the distinctions between patient groups categorized by low, moderate, or high CVRS risk.

We used factors produced in PCA as variables for logistic regression analysis in order to test its predictive capability towards CVRS high-risk values (≥4 points). Results are presented in Table 5.

The most important factor predicting high CVRS is Factor 3, which included uric acid, creatinine, and TAS.

## 3. Discussion

To our knowledge, this is the first study to examine redox status in an adolescent population in relation to cardiovascular risk. Also, this is the first study that examined composite scores of pro-oxidants (i.e., TOS, AOPP, and MDA) and antioxidants (i.e., TAS and SOD), as well as overall oxidative stress in an adolescent population, in order to obtain comprehensive insight into the disturbed redox homeostasis in high-risk individuals. While anti-oxy scores did not differ between CVRS subgroups, an increase in pro-oxy scores, as well as oxy scores, with CVRS risk increase was observed. This confirmed the relationship between CVRS and redox status parameters, even though these parameters were of a totally different nature. Namely, we have examined byproducts of lipid peroxidation, MDA; oxidative protein modification, AOPP; as well as a comprehensive indicator of all pro-oxidants, TOS. Indeed, not only were these biomarkers (MDA, AOPP, TOS) significantly higher in the higher-risk group compared to the low-risk group, but a significant positive correlation between these parameters and CVRS was confirmed. Although antioxidant enzymes SOD and TAS did not differ between CVRS risk subgroups, the TAS/TOS ratio representing antioxidant blood potency was significantly lower in both moderate- and high-CVRS risk groups compared to the low-risk group, indicating diminished antioxidant defense in adolescents with moderate and high cardiovascular risk.

Previous studies in adult populations confirmed the association between oxidative stress and CVD [16,18]. Even more so, in a prospective cohort of 774 adult patients with chronic heart failure, higher MDA levels could predict 1-year all-cause mortality [18], supporting the reliability of evaluation of oxidative stress biomarkers in CVD.

Regarding lipid parameters in our present study, adolescents in the high-risk group exhibited lower HDL-c than peers in the low-risk group. Also, adolescents in the moderate-risk group had higher TG levels as compared to those in the low-risk group, suggesting that atherogenic dyslipidemia could be present even at a young age. Atherogenic dyslipidemia is intensified by the presence of sdLDL and accelerates atherosclerosis [19]. Lower HDL-c and higher TG levels are also recorded in Brazilian teenagers with obesity aged 12–17 years as compared to normal-weight peers [20]. An increased oxidative stress and higher cIMT, an early sign of endothelial dysfunction, were shown in teenagers with obesity [20]. We have also previously confirmed higher cIMT in young women with obesity as compared to normal-weight counterparts, as well as a relationship between cIMT and CVRS in this cohort [21], suggesting increased cardiovascular risk even in young populations living with obesity.

The complex interrelationship between redox status homeostasis and obesity-related cardiometabolic disturbances in the present study was further explored. Namely, PCA showed grouping cardiometabolic and redox status parameters according to equal variability in several factors, i.e., pro-oxidant-dyslipidemia-related factor (i.e., AOPP, TG, and TOS), hepatic steatosis-related factor (i.e., ALT and AST), and antioxidant-renal function-related factor (i.e., uric acid, creatinine, and TAS). Among them, the antioxidant-renal function-related factor showed significant predictive capability towards CVRS high-risk values.

Cardiomyocytes are the source of a large number of mitochondria for adenosine triphosphate (ATP) generation via oxidative phosphorylation [22]. The underlying mechanism that affects CVD pathogenesis is mitochondrial dysfunction with consequent uncoupling of the electron transport chain and diminished energy supply. The increased production of ROS via complexes I and III in myocytes, endothelial cells, and neutrophils, lead to inflammation and endothelial dysfunction, triggering cardiac hypertrophy, hypertension, atherosclerosis, and cardiomyocyte apoptosis [23]. Depending on the availability, the ATP is generated mainly via the beta-oxidation of fatty acids, but glucose can also be utilized as a source of energy [23]. The insulin signaling is disrupted in CVD, leading to a decrease in the ATP amount. The disrupted insulin signaling pathways, precisely compromised phosphatidylinositol 3-kinase (PI3K) pathways, triggered by ROS and adipocytokines secreted from visceral adipose tissue, promoting lipolysis in adipose tissue and increasing the flux of free fatty acids into the liver. An increased flow of free fatty acids enhances oxidative phosphorylation and lipid peroxidation, a process that accelerates hepatic steatosis [12]. De novo lipogenesis increases the synthesis of TG-rich very-low-density lipoproteins (VLDL), sdLDL, and small HDL particles and further aggravates oxidative stress and inflammation. Small HDL particles undergo fast catabolism, which leads to lower HDL-c levels [12]. On the other hand, increased activity of protein kinase C (PKC) and nicotinamide adenine dinucleotide phosphate (NADPH) oxidase inhibits endothelial nitric oxide synthase (eNOS), thus lowering the synthesis of NO in vascular smooth muscle cells and leading to vasoconstriction, dysfunction of the endothelium, and consequent subclinical atherosclerosis [24]. The cytotoxic effects of free fatty acids also favor the impairments of other organs, such as glomerular and tubulointerstitial injury, and if prolonged, precede renal disease [25], which was confirmed by the relationship between lipid parameters and kidney function biomarkers in previous studies [26,27].

The strength of the study is reflected in the fact that we included a relatively large number of adolescents and a wide range of redox status parameters. Most of the previous studies included both children and adolescents in the research [14,15,28], so the confounding factor of puberty was not excluded. We included high school students, adolescents narrowly aged 16–19 years, in whom the criteria for adult metabolic syndrome can be applied, thus avoiding variations in the different criteria for children under 16 years of age and hormonal disturbances [13]. Also, other potential factors that contribute to oxidative stress and inflammation were eliminated, such as medication use and alcohol consumption [29], since we excluded adolescents with these factors from the research.

The limitations of the study are its cross-sectional nature and relatively small sample size, as well as the inability to measure cIMT, oxidized LDL, and lipoprotein subclasses that might give deeper insight into the relationship between redox imbalance and CVRS. Also, the measurement of sex hormones could give a valuable insight into the sex-specific relationships between examined biomarkers and CVRS. Subsequent studies with larger sample sizes would be beneficial to overcome such limitations.

## 4. Materials and Methods

### 4.1. Study Population

The present study examined a cohort of 182 adolescents (76% girls). Each adolescent signed an informed consent confirming that they were well informed about the study and were willing to participate. Parents’ written consent for participants younger than 18 years was also provided. The teenagers filled in a questionnaire on lifestyle habits (i.e., smoking, alcohol use), illnesses, and medication use. Inclusion criteria were healthy adolescents aged 16–19 years who were motivated to participate. Adolescents with a sign of infection and high sensitivity C-reactive protein (hsCRP) ≥ 10 mg/L, as well as those with a history of alcohol consumption, were excluded from the examination.

All teenagers underwent venipuncture and anthropometric measurements for waist circumference (WC), body height, and body weight. The body mass index (BMI) was calculated. Systolic blood pressure (SBP) and diastolic blood pressure (DBP) were also measured. The study was approved by the Ethical Committee of Primary Health Care Center, Podgorica, Montenegro, and the research was conducted according to the Declaration of Helsinki principles.

### 4.2. Biochemical Analyses

The blood sampling was performed in the morning between 7 and 10 a.m., in the fasting state of at least 8 h. Serum levels of hsCRP, glucose, urea, creatinine, uric acid, total cholesterol (TC), high-density lipoprotein cholesterol (HDL-c), triglycerides (TG), low-density lipoprotein cholesterol (LDL-c), aspartate aminotransferase (AST), alanine aminotransferase (ALT), and total proteins were determined by a Roche Cobas c503 chemistry analyzer (Roche Diagnostics GmbH, Mannheim, Germany).

### 4.3. Oxidative Stress Biomarkers

#### 4.3.1. Superoxide Dismutase (SOD)

Plasma superoxide dismutase (SOD) activity was measured with Misra–Fridovich’s method modified in our laboratory. The method is based on the ability of the SOD enzyme to inhibit auto-oxidation of epinephrine in an alkaline medium. The absorbance maximum of the pink oxidized product is at 480 nm in an alkaline medium of bicarbonate buffer, pH = 10.2. Epinephrine concentration should be adjusted at a concentration that enables an absorbance change of 0.025 units per minute because this gives a condition for the highest inhibition of spontaneous epinephrine auto-oxidation. SOD activity is calculated as the percent of inhibition of epinephrine auto-oxidation [30].

#### 4.3.2. Total Antioxidative Status (TAS)

Total antioxidative status (TAS) was measured by a spectrophotometric method using 10 mmol/L ABTS [2, 2′-azino-bis (3-ethylbenzothiazoline-6-sulfonic acid)] as a chromogen [31]. The reduced ABTS molecule is oxidized to the ABTS^+^ radical cation using hydrogen peroxide in an acidic medium (acetate buffer 30 mmol/L pH 3.6). Upon defined conditions, dark green ABTS + molecules are stable for a long time of at least 6 months. Antioxidants present in the sample lead to reagent discoloration to a degree proportional to their concentrations. The reaction is calibrated with Trolox, and spectrophotometric measurement is performed at 660 nm, expressing the results in micromoles of Trolox equivalent/L.

#### 4.3.3. Advanced Oxidation Protein Products (AOPPs)

Advanced oxidation protein products (AOPPs) were assayed in a reaction mixture of 20 mM phosphate buffer pH 7.4, glacial acetic acid, and 1.16 M potassium iodide. The maximum absorbance of the built product is measured at 340 nm. Chloramine T was used as a standard, with a concentration range of 10–100 µmol/L [32].

#### 4.3.4. Malondialdehyde (MDA)

Malondialdehyde (MDA) concentrations were measured using the thiobarbituric acid-reactive substances (TBARS) employing 5 min incubation at 100 °C in order to obtain a pink-colored product, of which absorbance is then measured using spectrophotometry at 535 nm, as described in detail by Girotti [33]. The thiobarbituric acid reagent consisted of 15% trichloroacetic acid, 0.375% thiobarbituric acid, and 0.25 M HCl.

#### 4.3.5. Total Oxidative Status (TOS)

Total oxidative status (TOS) was measured by a spectrophotometric method optimized by Erel [34]. Oxidants of hydrogen peroxide and lipid hydroperoxide structures from the sample oxidized the ferrous ion–o-dianisidine complex to ferric ion. The reaction is performed in a high concentration of glycerol molecules (1.35 mol/L). The second reaction relies on the ferric ion reaction with xylenol orange (150 μmol/L) in an acidic medium, which makes a colored complex, measured at 560 nm. The assay is calibrated with hydrogen peroxide (water solution, 10–200 μmol/L), and the results are expressed in micromolar hydrogen peroxide equivalent per liter (μmol H_2_O_2_ Equiv./L).

AOPP, TOS, SOD, and TAS methods are implemented at the ILAB 300 plus analyzer in our laboratory (Instrumentation Laboratory, Milan, Italy).

#### 4.3.6. TAS/TOS Ratio

The TAS/TOS ratio was calculated as a representative of antioxidant blood potency.

### 4.4. Calculation of Pro-Oxidant, Antioxidant and Oxy Scores

In order to determine the oxidative score of each subject, the Z score statistics approach was used [35]. The mean value of the Z score of the measured pro-oxidant parameters is regarded as the pro-oxidant score, or pro-oxy score. The mean value of the Z score of the measured antioxidant markers is regarded as the antioxidant score, or anti-oxy score. The difference between the tested compound’s pro-oxidative and antioxidative scores is assumed to be the oxidative score, or oxy score.

### 4.5. Calculation of CVRS

CVRS was modified according to McMahan et al. [36] and was obtained by summarizing several risk factors: sex—female (points = −1), male (points = 0); fasting glucose (≥5.6 mmol/L, points = 5); low high HDL-c (<1.04 mmol/L, points = 1; between 1.04 and 1.55 mmol/L, points = 0; >1.55 mmol/L, points = −1); high non-HDL-c (<3.4 mmol/L, points = 0; between 3.4 and 4.0 mmol/L, points = 2; between 4.1 and 4.8 mmol/L, points = 4; between 4.9 and 5.6 mmol/L, points = 6; and >5.7 mmol/L, points = 8); obesity (BMI ≤ 30 kg/m^2^, points = 0 for both genders; BMI > 30 kg/m^2^, points = 6 for males); smoking (nonsmoker, points = 0; smoker, points = 1); and BP (BP < 130/85 mmHg, points = 0; high SBP ≥ 130 mmHg and/or DBP ≥ 85 mmHg, points = 4), as previously shown [4,6].

Participants were divided into low CVRS (i.e., −2 ≤ CV risk score ≤ 1), moderate (i.e., 2 ≤ CV risk score ≤ 4), and high CV risk score (i.e., CV risk score ≥ 5).

### 4.6. Statistical Analysis

The Kolmogorov–Smirnov test (for groups with ≥50 subjects) was used for data distribution analysis. For the intergroup comparison, Mann–Whitney U and Kruskal–Wallis tests were appropriate, according to the number of compared groups. The chi-square test was used for categorical data comparison. Correlation analysis was performed by using Spearman’s test. Factor analysis was applied as a principal components analysis (PCA) with the aim of grouping data (variables) according to equal variability and reducing the number of data, followed by univariate logistic regression analysis. A figure related to PCA biplot of patients subgroups according to CVRS risk (low, moderate, high) is generated by using free tool of Plotly Europe Ltd. (London, UK). A biplot is presented as multidimensional data that displays the relationships between variables in a two-dimensional plot. The principal component scores are represented by dots, and the loading vectors are represented by lines. The biplot presentation enables a clear visualization of the underlying structure of the data. The basic criterion for the existence of statistical significance is the *p* ≤ 0.05 (two-sided test).

## 5. Conclusions

An evident relationship between cardiovascular risk score and oxidative stress in the adolescent population supports the possibility for improvement in age-specific CVD risk algorithms by adding redox homeostasis parameters in addition to conventional ones. More studies are needed to enlighten the multifactorial interplay between obesity-related metabolic risk factors, oxidative stress, inflammation, and increased CVD risk in the adolescent population.

## Figures and Tables

**Table 1:** Basic sociodemographic, clinical, and biochemical parameters according to CVRS.

Parameter	CVRS			*p*
	Low Risk (−2–1) *n* = 114	Moderate Risk (2–4) *n* = 54	High Risk (≥5) *n* = 14	
Age (years)	16 (16–17)	17 (16–18)	17 (16–18)	0.070
Gender, m/f: *n* (%)	21/93 (18.4/81.6)	13/41 (24.1/75.9)	10/4 (71.4/28.6)	19.1, <0.001
Smoking status no/yes	106/8 (93.0/7.0)	49/5 (90.7/9.3)	10/4 (71.4/28.6)	6.8, 0.034
BMI (kg/m^2^)	22.1 (20.6–24.4)	23.3 (22.0–26.1) ***	27.0 (25.5–31.0) ***, #	<0.001
WC (cm)	72 (68–78)	76 (71–83) **	89 (75–100) ***, ##	<0.001
SBP (mmHg)	118 (114–125)	134 (130–137) ***	137 (134–144) ***, #	<0.001
DBP (mmHg)	74 (68–78)	82 (78–89) ***	81 (77–85) ***	<0.001
Glucose (mmoL/L)	4.7 (4.5–4.9)	4.7 (4.5–4.9)	4.9 (4.8–5.3) *,#	0.040
Urea (mmol/L)	3.8 (3.3–4.6)	3.7 (3.3–4.3)	3.8 (3.2–4.4)	0.620
Creatinine (µmol/L)	60 (53–71)	62 (57–67)	69 (62–80) **, #	0.006
Uric acid (µmol/L)	237 (212–288)	255 (218–294)	276 (231–331)	0.175
Total proteins (g/L)	77 (74–79)	78 (75–81)	78 (76–82)	0.224
hsCRP (mg/L)	0.40 (0.30–0.90)	0.70 (0.40–1.50) *	1.10 (0.40–4.80) **, #	0.013
TC (mmol/L)	4.01 (3.59–4.44)	4.09 (3.56–4.44)	3.84 (3.40–4.79)	0.729
HDL-c (mmol/L)	1.45 (1.32–1.68)	1.48 (1.20–1.73)	1.26 (1.02–1.44) *, #	0.033
LDL-c (mmol/L)	2.13 (1.80–2.54)	2.06 (1.84–2.53)	2.22 (1.81–2.77)	0.514
TG (mmol/L)	0.73 (0.57–0.88)	0.80 (0.67–1.01) *	0.73 (0.62–1.16)	0.037
TG/HDL-c ratio (mmol/L)	0.455 (0.366–0.568)	0.537 (0.375–0.791) *	0.608 (0.559–0.780) *	0.014

*p* from Kruskal–Wallis test; *, **, *** *p* < 0.05, 0.01, 0.001 vs. low-risk group, respectively; # *p* < 0.05, ## *p* < 0.01 vs. moderate-risk group according to Mann–Whitney U test.

**Table 2:** Redox status parameters change according to CVRS risk.

Parameter	CVRS			*p*
	Low Risk (−2–1) *n* = 114	Moderate Risk (2–4) *n* = 54	High Risk (≥5) *n* = 14	
SOD (U/L)	141 (131–146)	139 (28–146)	139 (137–142)	0.757
TAS (μmol/L)	1183 (1103–1241)	1176 (1111–1248)	1219 (1181–1265)	0.256
TOS (μmol/L)	6.5 (4.9–9.9)	7.8 (6.6–11.1) *	11.0 (5.8–12.2) *	0.005
TAS/TOS	176 (114–242)	142 (109–188) *	109 (98–196) *	0.015
AOPP (μmol/L)	12.7 (10.4–15.6)	12.7 (11.6–16.8)	15.5 (13.7–17.3) *	0.065
MDA (μmol/L)	2.56 (1.92–3.04)	2.89 (2.48–3.29) **	3.33 (2.89–4.00) ***, #	<0.001

*p* from Kruskal–Wallis test; *, **, *** *p* < 0.05, 0.01, 0.001 vs. low-risk group, respectively; # *p* < 0.05 vs. moderate-risk group according to Mann–Whitney U test.

**Table 3:** Correlation between CVRS and redox status parameters.

Parameter	CVRS (ρ, *p*)
TAS (μmol/L)	ns
TOS (μmol/L)	0.246, 0.001
SOD (U/L)	ns
AOPP (μmol/L)	0.231, 0.002
TAS/TOS	−0.208, 0.005
MDA (μmol/L)	0.339, <0.001

ρ, *p* from Spearman’s nonparametric correlation; ns—non significant.

**Table 4:** Factorial analysis in the group of healthy adolescents.

Factor	Variables	Factor Loadings	Variability Percentage (Total 41%)
Factor 1: Pro-oxidant-dyslipidemia-related factor	AOPP (µmol/L)	0.864	16
	TG (mmol/L)	0.784	
	TOS (µmol/L)	0.782	
Factor 2: Hepatic steatosis-related factor	ALT (U/L)	0.841	13
	AST (U/L)	0.800	
Factor 3: Antioxidant-renal function-related factor	Uric acid (µmol/L)	0.694	12
	Creatinine (µmol/L)	0.631	
	TAS (µmol/L)	0.631	

**Table 5:** Binary logistic regression analysis of PCA extracted factors as predictors for high CVRS risk.

Variables	B (SE)	Wald Coefficient	OR (95% CI)	*p*
Factor 1	0.408 (0.337)	1.461	1.503 (0.776–2.912)	0.227
Factor 2	0.073 (0.478)	0.024	1.076 (0.422–2.744)	0.878
Factor 3	1.034 (0.528)	3.839	2.810 (1.000–7.900)	0.050

SE—standard error, OR—odds ratio (95th CI—confidence interval); *p* from the binary logistic regression analysis.

## Data Availability

The datasets used and/or analyzed during the current study are available from the corresponding author (aleksandranklisic@gmail.com) on reasonable request.
